# Intraoperative ultrasonography in laparoscopic partial nephrectomy for intrarenal tumors

**DOI:** 10.1371/journal.pone.0195911

**Published:** 2018-04-26

**Authors:** Baolong Qin, Henglong Hu, Yuchao Lu, Yufeng Wang, Yang Yu, Jiaqiao Zhang, Zhongbiao Zhang, Hongbin Gao, Qing Wang, Shaogang Wang

**Affiliations:** 1 Department of Urology, Tongji Hospital, Tongji Medical College, Huazhong University of Science and Technology, Wuhan, China; 2 Department of Ultrasonography, Tongji Hospital, Tongji Medical College, Huazhong University of Science and Technology, Wuhan, China; Public Library of Science, UNITED KINGDOM

## Abstract

**Objective:**

To evaluate the feasibility and efficacy of intraoperative ultrasonography in laparoscopic partial nephrectomy (LPN) for intrarenal tumors.

**Patients and methods:**

All patients who underwent LPN for renal tumors in our institution from January 2010 to October 2016 were assessed retrospectively. Patients were divided into two groups, the first with totally intrarenal tumors (TIT group), defined as a solid renal mass with no exophytic element on both preoperative and intraoperative evaluations, and the second with exophytic tumors (control group). General information and perioperative data of the two groups were compared, including tumor characteristics, operative time, estimated blood loss, warm ischemia time and pathological findings. Intraoperative laparoscopic ultrasonography (ILUS) was used to precisely locate and delineate the TIT border, as well as seeking for other suspected lesions.

**Results:**

We identified 583 patients who underwent LPN in our center, including 46 in the TIT and 537 in the control group. All patients in the TIT group were evaluated by ILUS, and all TIT procedures were successfully performed with only one conversion to open surgery. The mean tumor sizes in the TIT and control groups were 2.42 ± 0.46 cm and 3.29 ± 1.43 cm (p < 0.001), respectively. The TIT group’s R.E.N.A.L. nephrometry score was higher than that of the control group (median 8.5 vs 6.0, p < 0.001), and their mean operation times were 127.2 ± 16.0 min and 120.1 ± 19.2 min, respectively. Mean estimated blood loss was higher in the TIT than in the control group (161.3 ml vs 136.6 ml, p = 0.003). Mean warm ischemia time differed in the TIT and control groups (22.2 ± 6.4 vs 20.6 ± 4.7 min, p = 0.105), but not significantly. Rates of open conversion and positive margins, as well as rates of major postoperative complications, pathological findings, and 1-month changes in renal function, were similar in the two groups.

**Conclusion:**

Intraoperative ultrasonography is technically feasible in patients undergoing LPN for TITs. This method may reduce the need for radical nephrectomy in patients with endogenic renal masses.

## Introduction

Compared with radical nephrectomy (RN), nephron-sparing surgery (NSS) has shown long-term survival benefits in patients with early-stage renal masses [1]. Laparoscopic partial nephrectomy (LPN) has become the standard for localized renal tumors in selected centers, with advantages of less invasive injury than open partial nephrectomy and more preservation of normal parenchyma than laparoscopic radical nephrectomy [2]. Despite these advantages, intrarenal tumors remain a major challenge for urologists since it cannot be visualized on kidney surface and often end up with a radical nephrectomy [3].

Intraoperative ultrasonography was first introduced for diagnostic purposes [4], followed by its use in real-time B-mode scanning for open surgery [5]. Intraoperative laparoscopic ultrasonography (ILUS) for screening has been accepted with the widely use of laparoscopic surgeries and it reveals additional diagnostic information [6]. In the present study, we report our experience of LPN with application of intraoperative ultrasonography in patients with totally intrarenal tumors (TIT).

## Patients and methods

This study was approved by the Ethical Committee of Tongji Hospital, Tongji Medical College, Huazhong University of Science and Technology (IRB ID: TJ-C20151224). Written informed consent was obtained from all patients.

### Patients

The clinical charts of all patients who underwent LPN for renal tumors from January 1, 2010 to October 31, 2016 were reviewed. Patients with cystic renal tumors were not included since the diagnosis and treatment of cystic renal tumors might differ from other solid renal tumors. Patients’ demographic characteristics, including age and sex, as well as preoperative clinical data, were recorded. All patients were evaluated preoperatively by conventional ultrasound and contrast-enhanced CT/MRI. R.E.N.A.L. nephrometry score [7] was used to assess the anatomical characteristics of renal tumors. Intraoperative data were collected, including tumor size and location, operation time, estimated blood loss, warm ischemia time (WIT), open conversion rate and radical nephrectomy rate. Postoperative data included pathological results, changes in renal function and the occurrence of major complications, including late bleeding, urine leakage and pseudoaneurysm. Based on tumor location, patients were divided into two groups, a TIT group, consisting of patients with a solid renal mass presenting with no exophytic element on both preoperative and intraoperative evaluations, and a control group, consisting of patients with exophytic tumors.

### Intraoperative laparoscopic ultrasonography (ILUS)

All patients in the TIT group underwent ILUS using an Aloka Prosound α7 (Aloka Co., Ltd., Tokyo, Japan) ultrasound machine and a laparoscopic ultrasound probe (UST-5550T Ultrasound; Aloka) pre-sterilized using the low-temperature plasma method. The scanning frequency of the probe was 4.0 ~ 10.0 MHz, with a linear array to provide rectangular images. Tumor blood flow during surgery was evaluated by color Doppler ultrasonography.

### Surgical techniques

**Fig 1:**
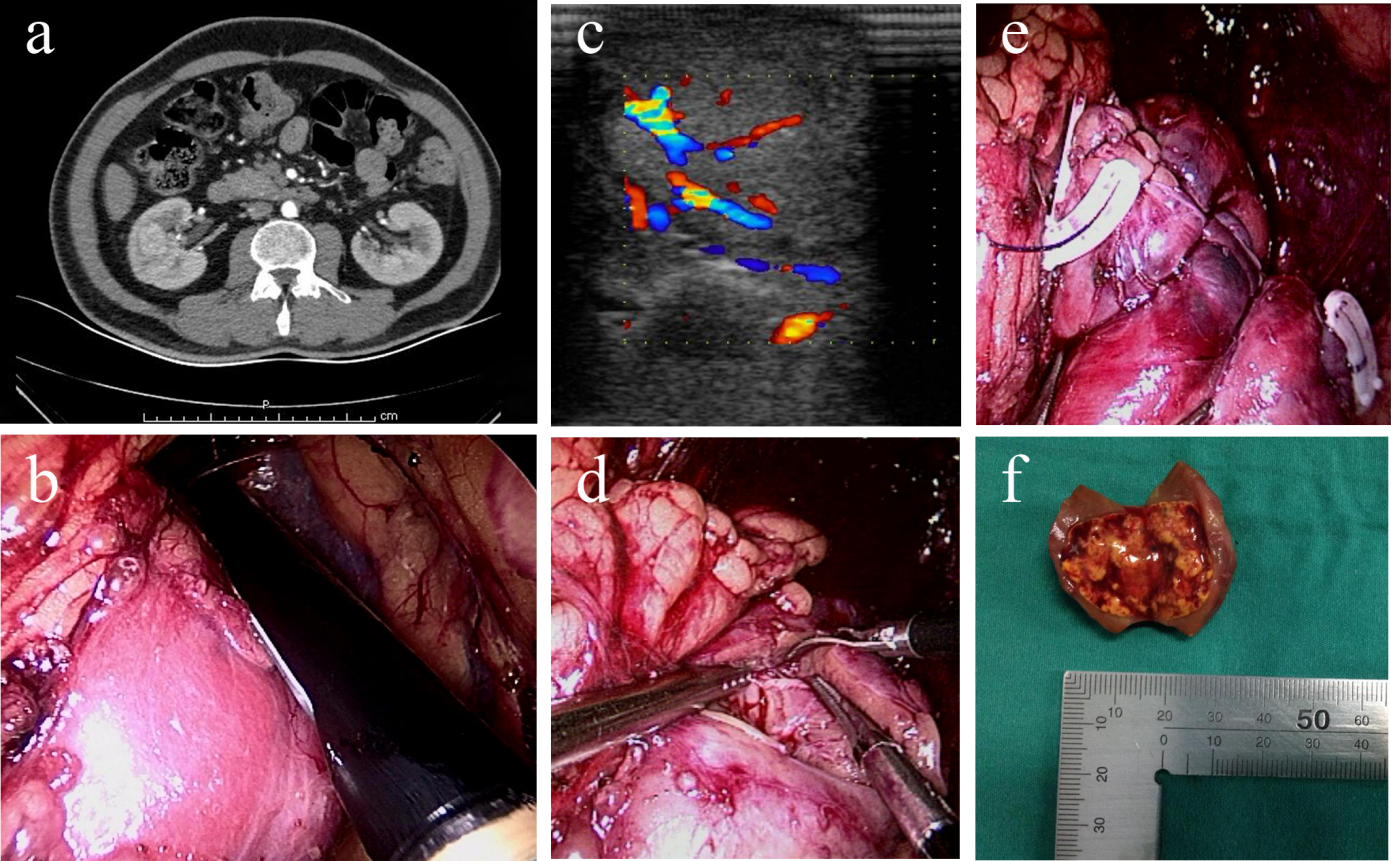
(a) Preoperative CT showing a TIT in right kidney. (b) Screen capture of ILUS with a probe scanning the kidney. (c) ILUS shows the TIT boundary and its blood flow distribution. (d) Laparoscopic invisible tumor resection after ILUS scanning. (e) Laparoscopic closure of the kidney crater with SRBS and hem-o-lok. (f) Excised specimen showing the integrity of TIT. (Informed consent for image publication was acquired).

All patients were placed in flank position with the ipsilateral side up after a satisfactory general anesthesia. A three-trocar access was established for the retroperitoneal approach. Gerota’s fascia was dissected along the anterior surface of the psoas muscle, and the kidney was exposed by a combination of blunt and sharp separation. The renal artery and vein were both dissected carefully using a vascular clamp. The Doppler probe was used to identify the renal artery if it was difficult to find. ILUS was performed to measure the tumor boundary and depth in the renal parenchyma, to estimate the distance from the collecting system and to search for potential satellite masses. Sometimes a electric monopolar hook was used to mark the tumor projection on the kidney surface circumferentially according to the ILUS images. Then the renal artery was clamped with a laparoscopic bulldog clamp, and ILUS was again performed to insure the artery blood flow was completely blocked. The tumor was incised with cold scissors along the margin rim of normal parenchyma. The distance from the cortical incision to the tumor was minimized, to ≤ 5 mm. The surgeon determined and adjusted the incision margin according to the ultrasonoscopy and his experience to insure excision along the tumor surface. Integrity of tumor capsule was checked after complete removal of the tumor by naked eyes without use of ultrasound. Renal repair was performed immediately with suture from the base of the crater. The incision was closed with running sutures or figure-eight stitches using 2–0 Vicryl or Quill self-retaining barbed suture (SRBS). If the tumor had infiltrated the collecting system, another running suture with 3–0 Vicryl or Quill SRBS was made from the incisal collecting system after incision of the tumor. A hem-o-lok clip was placed at the end of the suture. The bulldog clamp was subsequently removed to release blood flow. The artery blocking time (warm ischemia time) was controlled in 30 min, if possible, to ameliorate ischemia-reperfusion injury. Doppler scan was again performed to assess the restoration of blood flow or bleeding. Hemostatic gauzes or biological glue was applied to the excision margin. The excised specimen was placed in a laparoscopic removal bag and extracted. Finally, a perinephric drain was inserted into the retroperitoneum.(Fig 1)

### Statistical analysis

Continuous variables were expressed as mean ± standard deviation (SD) and compared using t-tests for independent samples. Data normality was tested using Shapiro–Wilk tests, with Welch’s adjustment applied for non-homogeneous distributions. Categorical variables were compared using chi-square tests. All data were analyzed by SPSS version 18.0 (SPSS Inc., USA), with P values < 0.05 considered statistically significant.

## Results

A total of 632 patients underwent LPN during the study period, with all operations performed by a single surgeon (SG Wang.). 49 patients were excluded mainly because the preoperative imaging data were not available to identify whether the tumor was TIT or not, and some cases due to missing data of postoperative eGFR examination. Data were available for 583 individuals, including 46 in the TIT group and 537 in the control group. Table 1 shows the demographic and tumor characteristics of the patients in these two groups. Age, sex, tumor location and preoperative serum creatinine were similar in the two groups. Mean tumor size was significantly smaller in the TIT than in the control group (2.42 ± 0.46 cm vs 3.29 ± 1.43 cm, p < 0.001). The TIT group’s R.E.N.A.L. nephrometry score was higher than that of the control group (median 8.5 vs 6.0, p < 0.001).

**Table 1:** Patients demographics and tumor characteristics.

Variable	TIT group	Control group	P value
Number(n)	46	537	
Sex, n(%)			0.528
Male	29 (63.0%)	363 (67.6%)	
Female	17 (37.0%)	174 (32.4%)	
Mean (SD) age, years	51.7 (7.3), 36 ~ 74	53.8 (11.6), 21 ~ 82	0.075
Sides			0.731
Left	24	266	
Right	22	271	
Mean (SD) tumor size, cm	2.42 (0.46)	3.29 (1.43)	< 0.001
Median (IQR) R.E.N.A.L. nephrometry score	8.5 (8, 9)	6 (5, 7)	< 0.001
Mean (SD) preoperative, eGFR mL/min/1.73 m2	88.6 (13.7)	85.1 (13.8)	0.098

eGFR, estimated GFR.

Table 2 shows the intraoperative and postoperative data of the TIT and control group. Mean operation time was significantly longer (127.2 ± 16.0 min and 120.1 ± 19.2 min, p = 0.016), and mean blood loss significantly greater (161.3 ml vs 136.6 ml, p = 0.003), in the TIT than in the control group. In contrast, WIT (22.2 min vs 20.6 min, p = 0.105), postoperative hospital stay (7.5 d vs 7.2 d, p = 0.376), and eGFR change at 1 month postoperation (-8.8 mL/min/1.73 m2 vs -8.4 mL/min/1.73 m2, p = 0.578) were similar in the TIT and control groups.

**Table 2:** Intraoperative and postoperative data of the TIT group and control group.

Variable	TIT group	Control group	P value
Mean (SD) operation time, min	127.2 (16.0)	120.1 (19.2)	0.016
Mean (SD) WIT, min	22.2 (6.4)	20.6 (4.7)	0.105
Mean (SD) blood loss, mL	161.3 (57.3)	136.6 (53.5)	0.003
Mean (SD) postoperative hospital stay	7.5 (2.3)	7.2 (1.1)	0.376
Major complications, n %	7/46 (15.2)	41/537 (7.6)	0.129
RN conversion rate, n %	4/46 (8.7)	24/537 (4.5)	0.354
Open conversion rate, n %	1/46 (2.2)	14/537 (2.6)	0.999
Positive surgical margin rate, n %	0/46 (0.0)	16/537 (3.0)	0.627
Mean (SD)% change in eGFR at 1-month postoperation	-8.82 (4.63)	-8.38 (5.21)	0.578

eGFR, estimated GFR.

Rates of major complications (Clavien 3 and 4) were similar in the TIT and control groups. No intraoperative complications were associated with the use of the laparoscopic ultrasonic probe. Four cases (8.7%) of TIT group were converted to RN, three of that owing to giant tumor adjacent to the renal hilum and one owing to multiple distant neoplasms. In contrast, the RN conversion rate of the control group was 4.5% (24/537). The open conversion rate was similar in the two groups (2.2% and 2.6%, respectively).

There were no differences between two groups in pathological types. Pathologic examination confirmed malignancy in 84.8% (renal cell carcinoma in 80.4% and leiomyosarcoma in 4.4%) of patients in the TIT group compared with 86.6% (renal cell carcinoma in 78.2%, leiomyosarcoma in 4.7% and others 3.7%) in the control group. Renal clear cell carcinoma, papillary carcinoma, and chromophobe cell carcinoma were the most common types of malignant tumors. Surgical margins were negative for all 46 TIT patients, while the control group’s positive margin rate was 3.0%. Close follow-up was recommended in all positive margin patients. We met two local recurrence patients with LPN at six months and one year postoperation and performed radical nephrectomy.

## Discussion

The surgical principle of renal masses is complete removal of the tumor while preserving as much of the remaining kidney as possible [8]. Compared with radical nephrectomy, nephron-sparing surgery (partial nephrectomy) markedly reduces the incidence of renal dysfunction [9]. LPN shows comparable surgical, oncologic, and renal function outcomes as open partial nephrectomy, as well as shorter operation time and duration of hospitalization, resulting in lower rates of perioperative morbidity and complications [10]. Refinements in techniques and hemostasis have enabled surgeons to deal with more difficult situations in a minimally invasive manner [11, 12].

TIT creates considerable challenges regarding its nonvisibility in naked eyes as well as variation in intrarenal position. In addition, surgeons must be able to address complete tumor resection and renal reconstruction meticulously within limited WIT so that they often adopt laparoscopic radical nephrectomy [3]. And even open partial nephrectomy for TIT is considered to be a technically difficult procedure with increased complication rates compared to that for peripheral renal tumors [13]. Chen et al [14] designed a new surgical planning and manual image fusion based on 3D model of renal tumors and the procedure facilitated visible-imaging-guided tumor resection in LPN for the resection of intrarenal tumors. In our study, the higher R.E.N.A.L. nephrometry score in TIT group’s showed more complexity and this selected group of TIT was performed skillfully by a single experienced surgeon after his achievement of more than 100 LPNs for renal masses.

Given the complexity of TIT, our results showed the safety and feasibility of LPN with intraoperative ultrasound for this circumstance. Overall complication rates were similar in the 46 patients with TIT who underwent laparoscopic intraoperative ultrasound as in the control group. The risk of major complications, such as late bleeding and urine leakage, was no higher in the TIT than in the control group. The WIT and change in renal function, as evidenced by eGFR change at 1 month postoperatively, did not differ significantly in these two groups. The operation time was longer in the TIT group, perhaps because of the addition of intraoperative ultrasound and the need to suture the remaining renal crater. The estimated blood loss was higher in the TIT group, as these tumors were deeper, requiring incision of additional parenchyma and blood vessels. Only four patients required radical nephrectomy and only one required conversion to open surgery, with no patient in the TIT group having positive surgical margins.

WIT in both groups were within the reasonable range to minimize ischemia-reperfusion injury [15]. Although WIT was somewhat longer in the TIT than in the control group, the differences was not statistically significant. The extra few minutes of WIT in the TIT group may be related to the implementation of US as well as deep excision and suture of tumors. Our mean WIT of TIT was shorter than in previous studies and it decreased gradually as the number of cases increased [3, 16].

One of the keys to our success is the use of intraoperative ultrasound. Intraoperative real-time ultrasound had been proved of value, particularly in hepatobiliary and pancreatic surgeries since its arising in early 1980s [17]. ILUS can provide higher ultrasonic frequencies to acquire better images compared with conventional transcutaneous US [18]. In our study, a flexible laparoscopic ultrasound probe was used to accurately determine tumor boundaries and depth within the parenchyma, as well as to assess the distance from the collecting system. ILUS in this study was performed by a senior sonographer with good knowledge of urological anatomy. After scanning, the renal artery was completely blocked to reduce the risk of follow up bleeding. The surgeon followed the incisal margins specified by the sonographer, after which the tumor was cut integrally using cold scissors.

Chung et al [3] reported 55 TIT cases of 800 patients who underwent LPN patients in 2011 which concluded LPN for TIT was technically effective and safe. Similarly, Nadu et al [16] evaluated outcomes of LPN in 41 patients with TIT and the results revealed that TIT should not as a definitive indication for RN. Our study affirmed that, when combined with intraoperative ultrasound, LPN was safe and feasible for TIT. Our techniques differed in two major aspects from those in previous studies. First, we utilized retroperitoneal rather than transperitoneal laparoscopic partial nephrectomy. The retroperitoneal approach is more popular in China than in western countries. Although the working space is limited, the retroperitoneal route provides direct access to the perinephric space with little bowel mobilization [19]. Retroperitoneal LPN was associated with a shorter operating time, a lower estimated blood loss, and a shorter hospital stay than transperitoneal technique [20]. Second, we utilized a running SRBS during closure of the kidney crater. The use of SRBS for renal repair is safe and can also significantly reduce WIT as well as bleeding risk [21]. To further reduce WIT, we placed a hem-o-lok clip at the end of the sutures. And no obvious postoperative bleeding was found in support of our technique.

The limitations of our study are mainly related to its retrospective nature and the analysis is based on only one surgeon’s experience. The number of TITs is relative small, suggesting the need for future large size studies.

## Conclusion

In conclusion, this study showed that, despite its inherent challenges, LPN for TIT is a safe and feasible procedure when combined with intraoperative ultrasonography. There were no significant differences between the TIT and exophytic tumor (control) groups in WIT, intraoperative and postoperative complication rates, postoperative hospital stay and positive margin rate. The effective use of intraoperative laparoscopic ultrasound provides accurate and important information and cover the shortage of LPN for TIT.

## Supporting information

S1 File Approval document from ethics committee. (PDF)

S2 File Patients’ information of TIT group. (XLSX)

S3 File Patients’ information of control group. (XLSX)
