# Supplementary material for: Intraoperative ultrasonography in laparoscopic partial nephrectomy for intrarenal tumors
Source: PLoS One. 2018 Apr 26;13(4):e0195911. doi: 10.1371/journal.pone.0195911 (PMC5919508; doi:10.1371/journal.pone.0195911)
Supplement: S1 File — (PDF) [file pone.0195911.s001.pdf]

华中科技大学同济医学院附属同济医院伦理委员会批准件

Tongji Hospital, Tongji Medical College，Huazhong University of Science and Technology
Institutional Review Board Approval
批准号 IRB ID：TJ-C20151224

项目名称<br>Project Name	术中超声在内生型肾肿瘤腹腔镜肾部分切除术中的应用<br>Intraoperative Ultrasonography in Laparoscopic Partial Nephrectomy for Intrarenal Tumors		
申请科室<br>Applicant Department	泌尿外科<br>Department of Urology		
项目负责人<br>Principal Investigator	王少刚<br>Shaogang Wang	职称<br>Title	主任医师<br>Chief Physician
报送材料<br>Submitted Materials	课题研究方案<br>Research Protocol	有/Yes■无/No☐	
	观察记录表<br>Observation Records	有/Yes■无/No☐	
	研究人员名单<br>Researchers List	有/Yes■无/No☐	
审查<br>Review Items	研究者资格<br>Researchers Qualifications	符合要求/Qualified ■<br>不符合要求/Not Qualified ☐	
	课题研究方案<br>Research Protocol	适合/Appropriate ■<br>不适合/Not Appropriate ☐	
有效期<br>Valid Date	2016 年 1 月至 2016 年 12 月<br>From 01/2016 to 12/2016		

评审意见/ Review Approval:

王少刚的上述研究涉及到受试者的病情隐私。经华中科技大学同济医学院附属同济医院医学伦理委员会审议研究方案及知情同意书，一致认为该研究方案设计符合《赫尔辛基宣言》原则，充分尊重受试者及其家属的知情同意权。研究过程中使用的实验手段先进、科学，采样过程符合医疗常规，并制定了受试者保护方案。

The Shaogang Wang’ study concerning the client's right to privacy. The study was approved by the Ethical Committee of Tongji Hospital, Tongji Medical College, Huazhong University of Science and Technology and was conducted according to the principles of the Declaration of Helsinki. Written informed consent was obtained from the subject, and his study considered Declaration of Helsinki as a statement of ethical principles.

华中科技大学同济医学院附属同济医院伦理委员会
Ethical Committee of Tongji Hospital, Tongji Medical College,
Huazhong University of Science and Technology
日期/Date: 24/12/2015
